# Sexual Health and Sexually Transmitted Infections in the North American Arctic

**DOI:** 10.3201/eid1401.071112

**Published:** 2008-01

**Authors:** Dionne Gesink Law, Elizabeth Rink, Gert Mulvad, Anders Koch

**Affiliations:** * University of Toronto, Toronto, Ontario, Canada; † Montana State University, Bozeman, Montana, USA; ‡ Centre for Primary Health Care, Nuuk, Greenland; § Statens Serum Institut, Copenhagen, Denmark

**Keywords:** Arctic, aboriginal health, sexual health, sexually transmitted diseases, community-based participatory research, perspective

## Abstract

Our objective was to describe the basic epidemiology of sexually transmitted infections for Arctic and sub-Arctic regions of North America. We summarized published and unpublished rates of chlamydial infection and gonorrhea reported from 2003 through 2006 for Alaska, Canada, and Greenland. In 2006, Alaska reported high rates of chlamydial infection (715 cases/100,000 population) compared with the United States as a whole; northern Canada reported high rates of chlamydial infection (1,693 cases/100,000) and gonorrhea (247 cases/100,000) compared with southern Canada; and Greenland consistently reported the highest rates of chlamydial infection (5,543 cases/100,000) and gonorrhea (1,738 cases/100,000) in the Arctic. Rates were high for both men and women, although the highest incidence of infection was predominantly reported for young women in their early twenties. We propose that community-based participatory research is an appropriate approach to improve sexual health in Arctic communities.

## Abstract 2

STI rates reported for the Arctic are much higher than those reported for their southern counterparts.

Four million people live in the Arctic (*1*), yet little is known about sexual health and sexually transmitted infections (STIs) in the circumpolar North. Arctic communities in North America comprise a large proportion of Native American, First Nation, Metis, Inuit, and other aboriginal peoples living in harsh climates, diverse landscapes, and a variety of community structures including urban, micropolitan, reserves or reservations, towns, villages, settlements, and remote fly-in communities. Access to healthcare varies by community and country and patient concerns about the preservation of confidentiality remain a barrier to accessing healthcare.

STI intervention and prevention strategies have been developed primarily for urban and suburban environments (*2*,*3*), the rural South (*4*–*7*), Latino communities (*8*), and developing countries, primarily in Africa (*9*,*10*). Cultural differences alone will affect their generalizability to communities in the Arctic. This is further emphasized by Bjerregaard et al. (*11*) who stated: “Intervention models developed under quite different circumstances cannot be expected to work in Greenland and intervention studies are highly needed.” However, combining the global knowledge gained from previous interventions involving other populations with the local knowledge and infrastructure of Arctic communities is important to develop innovative, culturally appropriate, and sustainable STI intervention strategies.

Our objective was to describe STI trends in the circumpolar Arctic, focusing on the North American continent (United States, Canada, and Greenland). We also propose a community-based participatory research approach to conducting research and planning interventions involving Arctic communities.

## Methods

Data on chlamydial infection and gonorrhea in the United States, Canada, and Greenland were collected from a variety of sources. Rates for the United States and Alaska were obtained from federal (*12*) and state (*13*) reports. Rates for Canada were obtained in collaboration with the Public Health Agency of Canada STI Surveillance and Epidemiology Section (Public Health Agency of Canada, unpub. data; see also [*14*] for published summaries). Data reported for Canada’s northern territories (Yukon, Northwest Territories, and Nunavut) were combined into 1 statistic and compared to data reported for Canada’s southern provinces, which were also combined into 1 statistic. Data for Greenland were obtained from the Office of the Chief Medical Officer in Greenland (*15*,*16*) and compared with data reported for Denmark by the Statens Serum Institut (www.ssi.dk). In situations where STI rates were not already available (primarily Greenland and Denmark), rates were calculated by dividing the number of cases by the total population and multiplying by 100,000. Population estimates were obtained from the US Census Bureau, Statistics Canada, Statistics Greenland, and Statistics Denmark (StatBank).

Chlamydial infection and gonorrhea rates reported for the year 2006 were standardized by age and sex to the year 2000 US population so that rates could be compared across countries after correcting for age and sex differences between the different populations. Rates were standardized to the year 2000 US population by using age- and sex-stratified counts available from the US Census Bureau (www.census.gov). Additionally, chlamydial infection and gonorrhea rates were stratified by the basic demographic characteristics of age, sex, and race (when available) to gain insights into target populations for community interventions. Rates by race were only available for Alaska.

## Results

As expected, chlamydial infection was the most highly reported STI for the United States, Canada, and Greenland (Table 1). Compared with other states in the United States, Alaska reported the highest rates of chlamydial infection in 2003 and second highest in 2004 and 2005 (*12*). Canada’s northern territories consistently reported the highest rates of chlamydial infection in Canada, which is consistent with the 1987–1994 rates measured by Orr and Brown (*17*) for the Keewatin District of the Canadian Central Arctic. Greenland reported chlamydial infection rates higher than Denmark and higher than any other country in the North American Arctic.

**Table 1:** Comparison of sexually transmitted infection rates reported for North America’s Arctic countries, 2003–2006

Yearly rate*	United States	Alaska, USA	Canada	Northern territories,† Canada	Southern provinces,† Canada	Denmark	Greenland
Chlamydial infection							
2003	301.7	601.1	189.4	1,433	185	342	3,255
2004	316.7	609.4	197.1	1,805	195	401	3,208
2005	332.5	664.4	200.4	1,952	195	441	4,762
2006	347.8	682	202.2	1,922	197	458	4,527
2006 standardized‡	470.9	715	205	1,693	200	681	5,543
Gonorrhea							
2003	115.2	88.3	26.0	264	25	3.5	1,162
2004	113.5	87.4	28.9	215	29	7.7	1,148
2005	115.6	91.5	27.8	212	28	8.2	1,350
2006	120.9	95	33.1	281	32	7.5	1,418
2006 standardized	164.4	101	33	247	32	6.5	1,738

*Per 100,000 population. Data from Centers for Disease Control and Prevention, 2006 (*12*); Public Health Agency of Canada, 2007 (*14*); Office of the Chief Medical Officer in Greenland (*15*,*16*); Statens Serum Institute surveillance Epi-data online (www.ssi.dk). †Canadian Northern territories: Yukon Territory, Northwest Territories, and Nunavut; Southern provinces: British Columbia, Alberta, Saskatchewan, Manitoba, Ontario, Quebec, Newfoundland, Nova Scotia, New Brunswick, and Prince Edward Island. ‡2006 standardized estimates are directly standardized to the year 2000 US population distributed by age and sex.

Co-infection with chlamydial infection and gonorrhea is common so we expected gonorrhea rates to be high for the Arctic regions. However, Alaska reported some of the lowest gonorrhea rates in the United States (*12*). As expected, however, the Canadian Northern Territories reported higher gonorrhea rates than their southern counterparts, and again, Greenland reported gonorrhea rates higher than those in Denmark and in any other country in the North American Arctic (Table 1).

Chlamydial infection rates reported for women were much higher than rates reported for men in Alaska (Table 2), Canada (Table 3), and Greenland and Denmark (Table 4). Compared to gonorrhea rates reported for men, however, gonorrhea rates were higher for women <30 years of age in Alaska (Table 2), <20 years of age in Canada (until 2006, when rates remained higher for women <24 years of age; Table 3), and <20 years of age or <30 years of age for women in Greenland (Table 4). Gonorrhea rates reported for men in Denmark were consistently higher than rates reported for women (Table 4). Reported rates of chlamydial infection and gonorrhea were consistently high for both men and women 15–30 years of age, particularly for those 20–24 years of age, regardless of country.

**Table 2:** Chlamydial infection and gonorrhea rates per 100,000 population by age, sex, and race reported for Alaska, 2006*

Characteristic	Chlamydial infection rates			Gonorrhea rates	
	M	F		M	F
Age, y					
15–19	966	4,158		78	346
20–24	2,673	4,990		344	496
25–29	1,250	2,253		185	309
30–34	607	854		162	162
35–39	269	420		134	81
Race					
White	235	389		28	41
Alaska Native/ American Indian	927	3,012		153	344

*Source (*13*).

**Table 3:** Chlamydial infection and gonorrhea rates per 100,000 population by age and sex reported for northern territories (NT) and southern provinces (SP) in Canada, 2004–2006*

Characteristic	2004						2005						2006				
	NT			SP			NT			SP			NT			SP	
	M	F		M	F		M	F		M	F		M	F		M	F
Chlamydia, age, y																	
<14	22	319		1	19		22	361		0.8	18		8	296		1	16
15–19	3,050	10,014		276	1,428		3,193	11,866		270	1,367		3,374	10,771		278	1,329
20–24	4,778	9,408		695	1,478		5,255	8,893		701	1,470		4,982	9,431		703	1,475
25–29	3,154	4,492		405	552		3,623	4,435		423	562		3,192	5,024		419	592
30–39	1,292	1,913		141	158		1,461	1,856		157	158		1,697	1,812		164	170
40–59	338	359		31	22		486	450		34	21		399	432		36	24
>60	120	142		4	1		90	215		4	1		84	126		5	2
Total	1,190	2,451		128	260		1,339	2,595		132	256		1,312	2,556		134	258
Gonorrhea, age, y																	
<14	0	23		0	2		7	38		0	3		0	23		0	4
15–19	376	761		57	124		437	737		53	112		671	1,473		63	132
20–24	820	763		126	99		968	650		118	102		1,038	1,246		132	118
25–29	738	332		91	43		689	433		93	41		557	526		104	57
30–39	306	169		65	14		311	86		61	15		267	234		67	21
40–59	137	15		23	3		71	45		23	2		86	25		27	0
>60	24	0		4	0		22	0		4	0		42	25		5	0
Total	240	189		37	21		239	184		35	20		251	312		40	25

*See (*14*).

**Table 4:** Chlamydial infection and gonorrhea rates per 100,000 population by age and sex reported for Greenland (GLD) and Denmark (DK), 2004–2006

Characteristic	2004						2005						2006				
	GLD			DK			GLD			DK			GLD			DK	
	M	F		M	F		M	F		M	F		M	F		M	F
Chlamydia, age, y																	
15–19	9,378	20,332		944	3,361		7,986	31,383		1,243	3,891		12,462	27,125		1,345	4,095
20–24	13,229	16,890		1,966	3,526		9,003	20,594		2,264	3,720		17,154	21,854		2,391	3,768
25–29	6,444	8,590		978	1,284		5,776	11,006		1,089	1,322		10,837	10,445		1,114	1,335
>30	916	1,229		86	41		805	1,005		85	87		1,630	1,507		93	88
Total	2,481	4,158		287	511		3,852	5,597		324	554		3,704	5,468		343	571
Gonorrhea, age, y																	
15–19	3,714	7,346		13.9	7.7		2,141	7,801		11.5	4.0		5,360	8,763		11.1	6.5
20–24	4,663	4,450		46.4	7.4		2,993	5,648		39.9	13.1		5,994	5,858		32.5	16.0
25–29	3,056	1,795		51.3	4.5		1,984	3,184		44.9	4.7		3,755	2,374		39.4	10.8
>30	493	434		10.7	1.2		224	312		14.1	1.1		363	498		12.4	1.2
Total	1,047	1,301		13.6	1.9		1,174	1,550		14.6	2.0		1,252	1,609		12.5	2.6

## Discussion

Chlamydial infection rates were higher for Arctic and sub-Arctic areas in North America than for their southern counterparts. Gonorrhea rates reported for northern Canada and Greenland were also much higher than for their southern counterparts, although rates reported for Alaska were not very high. In 1741, Hans Egede, the first missionary to Greenland noted that “It is strange. . . that even though [Greenlanders] have free intercourse with other people, these are not infected” (*11*). However, for the past several years Greenland has reported chlamydial infection rates ≈10× higher, and gonorrhea rates ≈100× higher, than rates reported for Denmark and the highest rates of both infections in the North American Arctic (Table 1).

Chlamydial infection and gonorrhea rates reported for the Arctic and sub-Arctic are very high for both men and women, although the highest incidence of infection is predominantly reported for young women in their early 20s (Tables 2–4). True rates could be higher than reported for a variety of reasons. As in other settings, asymptomatic infection is high for both men and women and can result in missed cases. How much knowledge exists in remote communities about STIs, their symptoms, and what to do if one suspects he or she has an infection is unclear. Even if a person suspects that he or she has an infection, accessing healthcare can be a challenge since many of the Arctic communities are remote fly-in communities with limited healthcare resources. Additionally, many Arctic residents spend their summers away hunting or whaling, usually at great distances from their communities, and certainly far away from a healthcare provider. Another barrier to care in small communities is the issue of confidentiality and the common perception that it can be breached easily. This results in delayed healthcare seeking or missed infections. Partner notification can also be hindered by cultural norms and taboos. For instance, in some communities, talking about something can be regarded as the same as wishing it upon the people. Therefore there can be a reluctance or even movement against talking about STIs or naming sexual contacts. Finally, reporting infections can become a challenge in an already overtaxed healthcare system with limited infrastructure.

STI rates are quite variable across the North American Arctic and sub-Arctic (Tables 1–4). Access to healthcare and reporting differences could explain some of the difference in rates. For instance, Greenland has universal healthcare. Canada has universal healthcare, but it differs for on-reserve and off-reserve aboriginal people. Alaska only has universal healthcare for indigenous people. These different healthcare coverage strategies could affect the healthcare-seeking behavior of the populations that live with them. Another nuance of northern rates is the small underlying populations from which cases arise. The addition of 1 new case can result in a large change in the rate of infection. Additionally, because no international surveillance system is in place to monitor STIs, the information collected is not standardized between the countries. For instance, in the United States, the only country that collects racial information as part of its surveillance program, chlamydial infection, gonorrhea, and syphilis rates reported for American Indians and Alaska Natives were recently reported to be 2–6× higher than rates reported for non-Hispanic whites (*18*). In Canada, STI rates are suspected of being higher for aboriginal people, but no data exist to confirm this hypothesis. In Greenland, 89% of the population is Kalaallit Inuit, making it of arguable importance to collect racial information for Greenland.

There is a dearth of research pertaining to the factors that contribute to sexual health and STIs among aboriginal people (*18*,*19*). Chlamydial infection and gonorrhea are major causes of ectopic pregnancy in the Canadian Arctic (*17*), and STIs are highest for Canadian aboriginal people 15–24 years of age (*20*). Westernization, culture, and identity have been suggested as possible factors influencing STI transmission among Inuit youth in northern Canada (*20*); however, research is still needed to provide evidence for this hypothesis. Most other studies have focused on HIV and AIDS (*19*,*21*).

In Greenland, much of the STI research has also focused on HIV/AIDs. HIV/AIDS came late to Greenland compared to the rest of Europe and has remained limited to a heterosexual, alcohol-abusing group of persons of low socioeconomic status living in 2 communities in western Greenland. One reason that HIV, unlike other STIs, has not become a widespread epidemic across Greenland is because the prevalence of needle sharing and men who have sex with men is limited, considerably affecting the modes of transmission (*11*). However, research on HIV transmission among heterosexual persons, as well as the increased risk for co-infection with STIs and HIV, suggests that chlamydial and gonorrhea rates in Greenland are a public health concern that warrant further investigation (*22*–*24*).

As suggested by Steenbeek et al. (*20*), colonization and westernization in the Arctic may be responsible for increased rates of STIs for Arctic communities. We further hypothesize that these factors are contributing to disparately high STI rates in the Arctic through individual, familial, social, cultural, and environmental domains. We also hypothesize that high STI rates may only be a marker of greater underlying public health concerns such as substance abuse, poor mental health, and the legacy of historic trauma.

### Implications for Future Research

We propose that community-based participatory research (CBPR) is an appropriate approach to address sexual health and STIs in the Arctic. Sexual and reproductive health data for aboriginal populations are often not reported in national surveillance and survey reports (*25*). Also, indigenous communities have historically been reluctant to participate in research projects because traditional research methods, which emphasize the researcher as “the expert,” have not engaged indigenous communities in designing and implementing research projects (*25*). CBPR has been identified as an effective and essential strategy for conducting research with indigenous peoples because of its emphasis on community participation to build ownership of research projects and community-based interventions as well as empowering the community to address its health disparities (*26*,*27*).

Several components of CBPR support its use as a methodologic framework for conducting research in aboriginal communities. First, CBPR engages aboriginal or indigenous people in full and equal partnership with those communities in efforts to observe and respect tribal sovereignty and the right to self-determination (*28*). Second, the growing interest in addressing the interrelatedness of historic trauma and health disparities in indigenous populations and the inherent complexities of unraveling the interconnected components and concepts related to historic trauma and health can best be understood by discussions and conversations with indigenous communities (*29*,*30*). Third, a legacy of harm from past research, as well as mistrust of researchers, warrants the use of CBPR as a means to ensure that all phases of a research project, from the development of research questions to research design and data collection methods to dissemination of results, have community input and approval (*26*,*31*). Fourth, CBPR provides a forum to ensure timely communication of research results to the community by using information dissemination mechanisms that best meet the community’s needs. Finally, the limited research on sexual health among indigenous populations primarily focuses on problem theory that provides insights into the predisposing, enabling, and reinforcing factors related to engagement in high-risk sexual behavior among aboriginal communities. However, emerging evidence in the field of aboriginal sexual health suggests that a risk-based approach to understanding sexual behavior in these communities not only has a narrow and negative focus, with scant opportunities for indigenous groups to capitalize on their strengths, but also is not congruent with indigenous cultural and social beliefs and historical experiences (*32*). CBPR, because of its collaborative nature, empowers community members to capitalize on the strengths and resources available in their community.

## Conclusion

The use of CBPR as a framework in which to conduct sexual health research with and among indigenous populations is a promising approach that joins the strengths and skills of researchers with local knowledge, wisdom, traditions, and resources. The CBPR approach is much like taking a Bayesian approach to study design, data collection, analysis, interpretation, dissemination, and follow-up. Researchers provide global (prior) knowledge that is then integrated and updated with local (likelihood) knowledge provided by the community to produce a more holistic model of health. This approach means that study designs can be more effective, data collection can be more accurate and complete, interpretation of the results can be more insightful and relevant, dissemination of the study results can be more efficient and translated at the appropriate level for the community by community members, and interventions can be more effective, culturally appropriate, and sustainable. Community involvement in the project can also help facilitate translation of the research findings into clinical and political practice.
